# Oncostatin M is overexpressed in skin squamous-cell carcinoma and promotes tumor progression

**DOI:** 10.18632/oncotarget.26355

**Published:** 2018-11-23

**Authors:** Marie Simonneau, Eric Frouin, Vincent Huguier, Cynthia Jermidi, Jean François Jégou, Julie Godet, Anne Barra, Isabelle Paris, Pierre Levillain, Sevda Cordier-Dirikoc, Nathalie Pedretti, François Xavier Bernard, Jean Claude Lecron, Franck Morel, Laure Favot

**Affiliations:** ^1^ LITEC, Université de Poitiers, Poitiers, France; ^2^ CHU de Poitiers, Poitiers, France; ^3^ Bioalternatives, Gençay, France

**Keywords:** oncostatin M, cytokine, skin cancer, tumor microenvironment, immunotherapy

## Abstract

Cutaneous squamous cell carcinoma (cSCC) is the second most common keratinocyte malignancy and accounts for 20% of skin cancer deaths. Cancer is closely related to inflammation, but the contribution of the tumor microenvironment to cSCC development is poorly understood. We previously showed that oncostatin M (OSM), a cytokine belonging to the IL-6 family, promotes normal keratinocyte proliferation and migration, skin inflammation, and epidermal hyperplasia, both *in vitro* and *in vivo*. Here, we show that OSM is overexpressed in human cSCC and is associated with type 1 immune polarization. *In vitro*, OSM induced STAT-3 and ERK signaling, modified the expression of genes involved in cytokine signaling, proliferation, inhibition of apoptosis, and immune responses, and promoted proliferation and migration of malignant keratinocyte PDVC57 cells. PDVC57 cells grafted in the skin of mice led to rapid cSCC development, associated with OSM expression by tumor-infiltrating neutrophils. Finally, the absence of OSM (OSM-KO mice) led to a 30% reduction of tumor size and reduced M2 polarization in the tumor microenvironment. Globally, these results support a pro-tumoral role of OSM in cSCC development and suggest that a new therapeutic approach targeting this cytokine could be considered.

## INTRODUCTION

Cutaneous squamous cell carcinoma (cSCC) is the second most common keratinocyte malignancy after basal cell carcinoma in the Caucasian population [1]. Its incidence rate increases over the years due to aging and increasing recreational sun exposure [2]. cSCC is usually localized and surgical excision is the primary treatment modality. However, 5% of cSCC may metastasize to lymph nodes or internal organs. Indeed, 20% of deaths due to skin cancers are related to cSCC [3] because of the absence of therapies for this subset of tumors [4].

Over the last ten years, accumulating evidence has shown that cancer development is closely associated with inflammation [5, 6]. Tumors are infiltrated by immune cells, which can promote or antagonize tumor development, depending on their phenotypic and functional polarization [7–9]. Tumor-infiltrating macrophages are among the major immune cells that compose the tumor microenvironment. They can be divided into two phenotypic subtypes, depending on the composition of the microenvironment (cytokines, chemokines, membrane phenotype, transcription factors, and matrix proteins [10–12]). Classically activated macrophages, or M1 macrophages, are induced by interferon γ (IFNγ) and/or lipopolysaccharide. They promote a type 1 immune response mediated by proinflammatory cytokines, such as IFNγ or IL-12, and are associated with anti-tumor activity. In contrast, the alternatively activated macrophages, or M2 macrophages, are induced by IL-4 and IL-13 and promote a type 2 immune response, which includes the production of IL-4 and protumor activity [13–16]. Few studies have focused on macrophage polarization in cSCC. The composition of polarized macrophages in the cSSC microenvironment is mixed, including both M1 and M2 macrophages which play a role in the early stages of carcinogenesis [17, 18].However, the mechanisms of macrophage polarization have not been fully elucidated, mostly because of the complexity of the cytokine-mediated signals on the tumor microenvironment [19, 20]. Pro-inflammatory cytokines can promote tumor development. For example, skin carcinogenesis is reduced in tumor necrosis factor α (TNFα) deficient mice, suggesting a role for this cytokine in the early stages of tumor development [21]. Tumor development is also delayed in IL-23 or IL-23 receptor-deficient mice in various mouse models of cSCC [22]. IL-23 is a cytokine that induces Th17 polarization. The Th17 cytokines IL-17 and IL-22 promote proliferation and migration *in vitro* and tumor growth *in vivo* of human non-melanoma skin cancers [23]. However, no study has focused on the impact of cytokines on the inflammatory response and immune polarization during cSCC development.

Oncostatin M (OSM) is a multifunctional cytokine which belongs to the gp130 family, so called because of its anti-proliferative effects on melanoma cell lines [24]. It is secreted by T cells, monocytes/macrophages, dendritic cells, and neutrophils and has pleiotropic activities in immunity, hematopoiesis, bone modelling, and inflammatory processes [25, 26]. Amongst the numerous pro-inflammatory effects on tissues, such as the chest, joints, liver, or bowel, we previously reported that OSM targets both human and murine keratinocytes [27–32]. OSM mediates activation of mitogen activated protein kinase (MAPK) and Janus kinase/signal transducer activator of transcription (JAK/STAT) signal transduction pathways [33]. This signal is induced through the only functional receptor expressed on keratinocytes, the type II OSM-receptor, composed of gp130/OSM receptor β (OSMRβ) subunits. *In vitro*, OSM enhances the expression of chemokines and S100 family antimicrobial peptides and strongly inhibits keratinocyte differentiation [29].

Since its discovery, other studies have reported either beneficial or detrimental effects of OSM on tumor development [34]. For example, OSM directly increases proliferation of Kaposi's sarcoma cells and prostate carcinoma cells [35, 36]. Moreover, OSM promotes malignancy of cervical squamous cell carcinoma by enhancing migration and invasion [37]. More recently, OSM has also been shown to render the tumor microenvironment more permissive by inducing M2 polarization [38, 39]. However, there is not yet data reporting OSM involvement in cSCC development.

Here, we characterized human cSCC immune-cell infiltrates and the cytokine microenvironment and identified higher OSM expression in cSCC than normal skin. We demonstrated a direct effect of OSM on cSCC signaling, gene expression (cytokine signaling, proliferation, apoptosis and immune response), migration, and proliferation in the transformed keratinocyte cell line PDVC57. We further confirmed the role of OSM on cSCC development using an *in vivo* model of cSCC in WT *versus* OSM-deficient mice. Overall, we demonstrate that neutrophil-derived OSM production is involved in cSCC progression and M2 polarization.

## RESULTS

### Human cSCC characteristics

The present study included 22 patients, 16 males and six females, aged from 53 to 97 years (mean 78.0 years) (Table 1). The sex ratio of our cohort is in line with the male-female ratio of cSCC which is around 1.7 [40]. For one patient, two tumors were excised and included in this study. Four patients were organ transplant receivers: three had kidney transplants, and one a liver transplant.

**Table 1:** Patient characteristics

**Age (years)**	78.0 (53-97)
**Sex (Male/Female)**	16 M – 6 F
**Location of tumors (n)**	
**Head and Neck**	18
**Face**	9
**Scalp**	8
**Neck**	1
**Arm**	2
**Hand**	1
**Foot**	1
**Transplant patient (n)**	4
**Kidney transplant**	3
**Liver transplant**	1
**Tumor size (cm)**	2.4 (0.7-8.0)
**Tumor differentiation (n)**	
**Well differentiated**	13
**Moderately differentiated**	8
**Poorly Differentiated**	2
**Presence of keratinization (n)**	20
**Clark level of infiltration (n)**	
**III**	3
**IV**	11
**V**	9
**Infiltration depth (in mm according Breslow)**	6.83 (0.7-19)
**Tumoral or epidermal ulceration (n)**	16
**Perineural swelling (n)**	2
**Tumoral embolus (n)**	0
**Incomplete excision (n)**	4
**Local recurrences (n)**	2
**Metastasis (n)**	0
**Death related to cSCC (n)**	0

Tumors were located mostly on the head (17 cases; 78.3%): nine cases on the face (39.1 %) and eight on the scalp (34.8 %). Other localizations included the arm (two cases), and the neck, hand, and foot (one case each). Tumor size ranged from 0.7 to 8.0 cm (mean 2.4 cm) and consisted of mostly well-differentiated squamous cell carcinomas (13 cases, 56.5%) and those that were moderately (eight cases, 34.8%) or poorly differentiated (two cases, 8.7%). Keratinization, epidermal ulceration, perineural swelling, and tumoral embolus were observed in 20 (87%), 16 (65.6%), two (8.7%), and zero cases, respectively. The tumors were mostly infiltrative, as only three were Clark level III (limited to the papillary dermis), whereas 11 were Clark level IV (reaching the reticular dermis) and nine, Clark level V (reaching the hypodermis). The mean infiltration depth was 6.83 mm (0.7 to 19 mm), according to Breslow criteria. Four tumors (17.4%) were initially incompletely excised and two recurred (8.7%). No patient developed metastases nor died from cSCC (Table 1).

**Figure 1:**
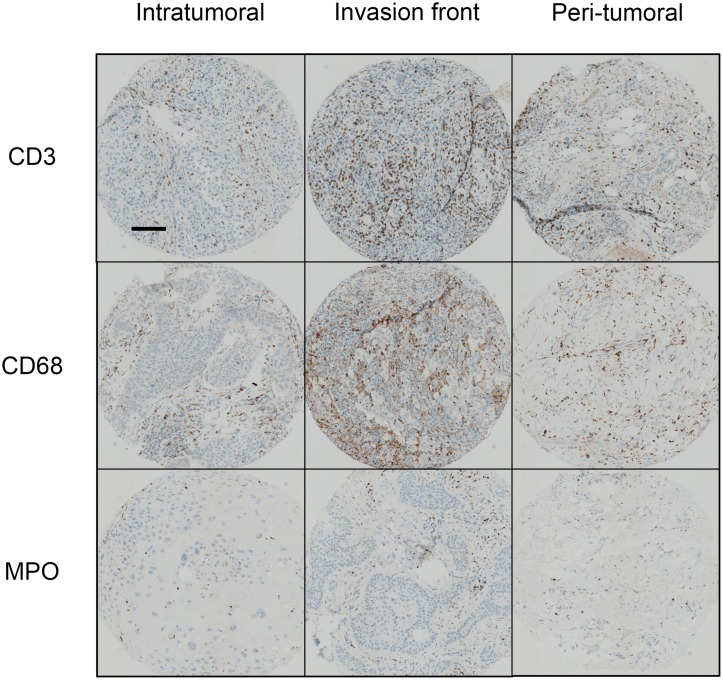
Immune infiltrates in human cSCC Human sample sections were fixed, embedded in paraffin, and stained with an anti-CD3, anti-CD68, or anti-MPO antibody. Scale bar: 100 μm. Quantification of inflammatory cells was performed using assisted counting software (Visilog Noesis).

Inflammatory infiltrates consisted mostly of T-cells (CD3^+^ cells) that predominated in the invasion front of tumors. Macrophages (CD68^+^ cells) were three times less abundant and polymorphonuclear cells (MPO^+^ cells) were rare but present in all tumors. Immune cells predominated at the invasion front and were only half as abundant in the tumor and peritumoral environment (Table 2) (Figure 1). Human cSCC presents a pro-inflammatory microenvironment.

**Table 2:** Immune infiltrates in human cSCC

	Intratumoral	Invasion front	Peritumoral
**CD3**	688 (88-2096)	1483 (142-3828) (^**^, p<0.01)	822 (50-3406)
**CD68**	330 (19-1102)	534 (143-1006) (^*^, p<0.05)	350 (94-770)
**MPO**	108 (0-707)	158 (14-676) (^**^, p<0.001)	66 (2-375)

Data are presented as the mean (min-max) number of inflammatory cells per field (0.6 mm diameter) determined using assisted-counting software (Visilog Noesis). We compared the cell mean number per field of CD3, CD68 or MPO positive cells in the intratumoral, invasion front, and peritumoral zones for each tumor. Statistical analysis were performed using a Kruskall-Wallis test with Dunn's Multiple Comparison Test ^*^p < 0.05, ^**^p < 0.01, ^***^p < 0.001.

**Figure 2:**
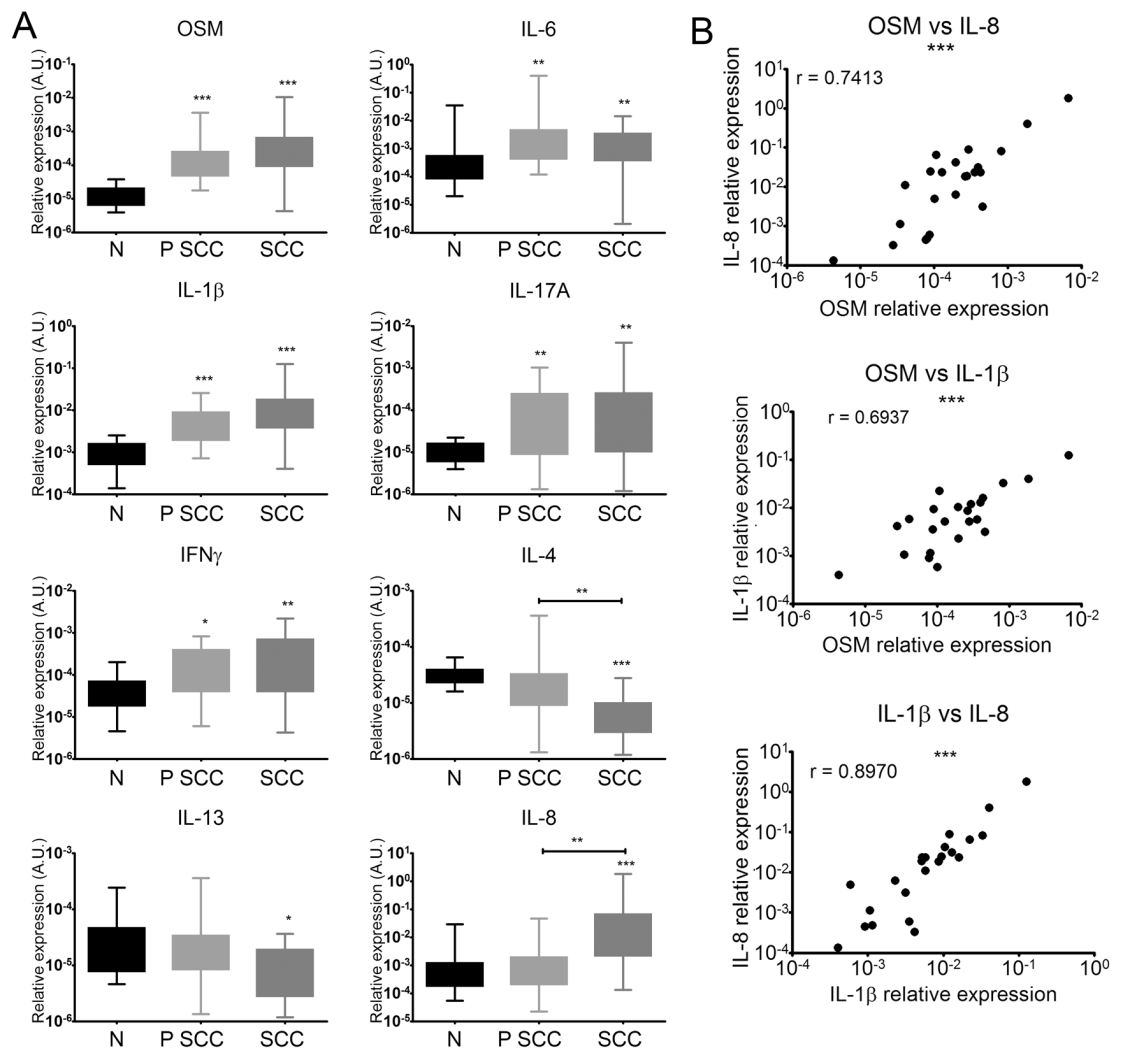
Human cSCC have a pro-inflammatory and predominantly Th1 immune microenvironment **(A)** Relative mRNA expression was measured by real-time qPCR for SCC (n = 23), perilesional tissues (P SCC) (n = 23), and normal skin (N) (n = 21). Data are presented as min to max box plots of relative mRNA expression. Kruskall-Wallis test with Dunn's Multiple Comparison Test ^*^p < 0.05, ^**^p < 0.01, ^***^p < 0.001. **(B)** Correlation between the relative expression of cytokines (OSM, IL-8, and IL-1β) of patient SCC lesions (n = 23). Data are presented as dot plots of relative mRNA expression. Spearman's correlation coefficients (r) are shown. ^*^p < 0.05, ^**^p < 0.01, ^***^p < 0.001.

We investigated the cytokine profile associated with human cSCC by quantifying mRNA levels of pro-inflammatory, type 1 and type 2 cytokines by RT-qPCR in the tumors (SCC), peritumoral skin (P SCC), and normal skin (N). There were significantly higher mRNA levels of the pro-inflammatory cytokines IL-6, IL-1β, IL-17A, and especially OSM in the perilesional tissues and SCC lesions than in normal skin (Figure 2A). IL-22 mRNA was undetectable in all cases, and IL-23A and TNFα mRNA expression was not modulated in perilesional tissues or SCC lesions relative to normal skin (data not shown).

The Th1 cytokine IFNγ was more highly expressed in perilesional tissues and SCC than normal skin (Figure 2A). In contrast, mRNA levels of the Th2 cytokine IL-4 were lower in perilesional tissues and even more so in SCC lesions, and IL-13 mRNA levels were lower in SCC lesions than in normal skin (Figure 2A). Finally, IL-8 expression was higher in SCC lesions than in normal skin and perilesional tissues (Figure 2A).

Moreover, OSM expression in tumor lesions strongly correlated with that of IL-1β (Spearman rank correlation (r) = 0.7413) and IL-8 expression (r = 0.6937) and the expression of the two cytokines correlated with each other (r = 0.8970) (Figure 2B).

### OSM induces STAT3 and ERK1/2 phosphorylation and modifies gene expression in PDVC57 cells

**Figure 3:**
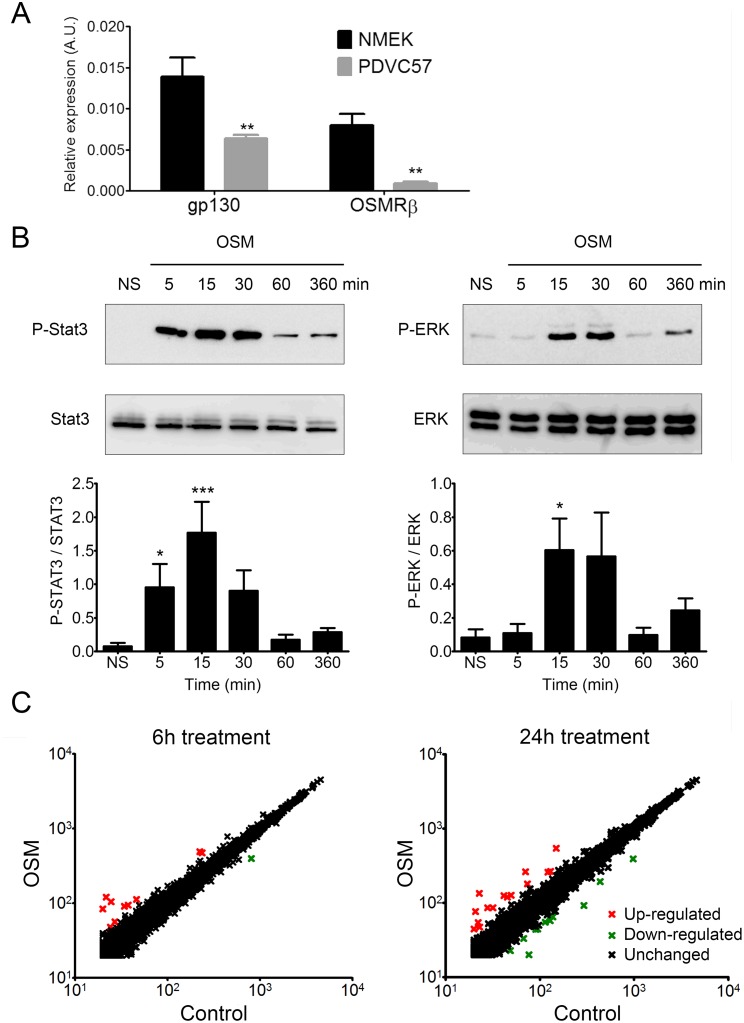
PDVC57 cells express functional OSMR type II involved in ERK1/2 and STAT3 signaling and modulation of gene expression **(A)** Relative mRNA expression of normal murine epidermal keratinocytes (NMEK) and PDVC57 cells cultured under normal conditions for 6 h was measured by real-time qPCR. Data are presented as the mean ± SEM of relative mRNA expression (A.U.). Two-way ANOVA test with Bonferroni post-tests, ^*^p < 0.05, ^**^p < 0.01. **(B)** PDVC57 cells were cultured with or without OSM (10 ng/mL) for 5 min, 15 min, 30 min, 1 h, or 6 h. Protein levels of P-STAT3, total STAT3, P-ERK 1/2 and total ERK were measured by western blotting. Data are presented as the mean ± SEM of phosphorylated to-total protein ratio for STAT3 or ERK from three independent experiments. Kruskall-Wallis test with Dunn's Multiple Comparison Test, ^*^p < 0.05, ^**^p < 0.01, ^***^p < 0.001. **(C)** Scatter-plot graphs representing the relative mRNA expression of PDVC57 cells stimulated, or not, with OSM (10 ng/mL) for 6 or 24 h. The X axis indicates the relative expression in the control and the Y axis the relative expression of OSM stimulated PDVC57 cells. The red dots reflect up-regulated genes and the green dots, down-regulated genes. Total RNA was isolated and labelled according to the Affymetrix protocol. Transcriptional profiles were obtained using Affymetrix GeneChip™ Mouse Gene 2.1 ST Array Plate.

The overexpression of OSM in human cSCC led us to question whether OSM has a direct role in cSCC development. We assessed the effect of OSM on malignant keratinocytes using the murine PDVC57 cell line [41]. PDVC57 cells expressed gp130 and OSMRβ, the two subunits of the type II OSMR, although at lower levels than normal murine primary keratinocytes (Figure 3A). However, OSM stimulation of PDVC57 cells induced strong and significant STAT3 phosphorylation from five to 30 minutes (Figure 3B), followed by phosphorylation of ERK 1/2 from 15 to 30 minutes (Figure 3B). We studied the PDVC57 gene expression profile under OSM stimulation by Affymerix microarray analysis (Figure 3C) and confirmed the modified gene expression by RT-qPCR (Table 3). Microarray analysis revealed OSM-dependent modulation of the expression of 11 genes six hours after OSM addition (10 overexpressed at least two-fold and one downregulated at least two-fold) and 26 genes (15 overexpressed, of which five were also upregulated after six hours of treatment, and 11 downregulated) after 24 hours of treatment.

**Table 3:** Effect of OSM on gene expression in PDVC57 cells

**6 h**	**Gene Symbol**	**Gene name**	**Fold change**
	Osmr	oncostatin M receptor	4.6
	Bcl3	B cell leukemia/lymphoma 3	3.9
	Il13ra1	interleukin 13 receptor. alpha 1	2.8
	Krt6a	keratin 6A	2.4
	Nek6	NIMA (never in mitosis gene a)-related expressed kinase 6	2.1
**24 h**	**Gene Symbol**	**Gene name**	**Fold change**
	Lcn2	lipocalin 2	20.3
	Osmr	oncostatin M receptor	5.9
	Bcl3	B cell leukemia/lymphoma 3	4.6
	Krt6a	keratin 6A	4.5
	Socs3	suppressor of cytokine signaling 3	4.2
	Urah	urate (5-hydroxyiso-) hydrolase	2.3
	Serpinb2	serine (or cysteine) peptidase inhibitor. clade B. member 2	2.1
	Il13ra1	interleukin 13 receptor. alpha 1	2.0
	Thbs1	thrombospondin 1	0.5
	Csf1	colony stimulating factor 1 (macrophage)	0.5
	Gfra2	glial cell line derived neurotrophic factor family receptor alpha 2	0.4

Fold changes were analyzed by qPCR and genes with a fold change < 2 or > 0.5 were selected.

The gene list was submitted for an analysis of functional annotation by Database for Annotation, Visualization, and Integrated Discovery (DAVID) software. The analysis showed that some genes are involved in cytokine signaling (OSMR, STAT3, IL13RA1, SOCS3), apoptosis (SOCS3, SERPINB2, BCL3, NGFR, THBS1, STAT3, ANGPTL4), proliferation (CSF2, OSMR, CSF1, AREG, THBS1, STAT3), macrophage differentiation and chemotaxis (CSF1, CSF2, THBS1), and the immune response (CSF1, CSF2, TLR3, NGFR, THBS1). Analysis by qPCR confirmed the up-regulation of five genes after six hours (OSMR, BCL3, IL13RA1, KRT6A and NEK6) and 11 genes were modulated after 24 hours of treatment (eight upregulated and three down regulated). Among the eight upregulated genes, four (OSMR, BCL3, IL13RA1, KRT6A) were already upregulated six hours after OSM addition (Table 3).

### OSM induces migration and proliferation of PDVC57 cells

**Figure 4:**
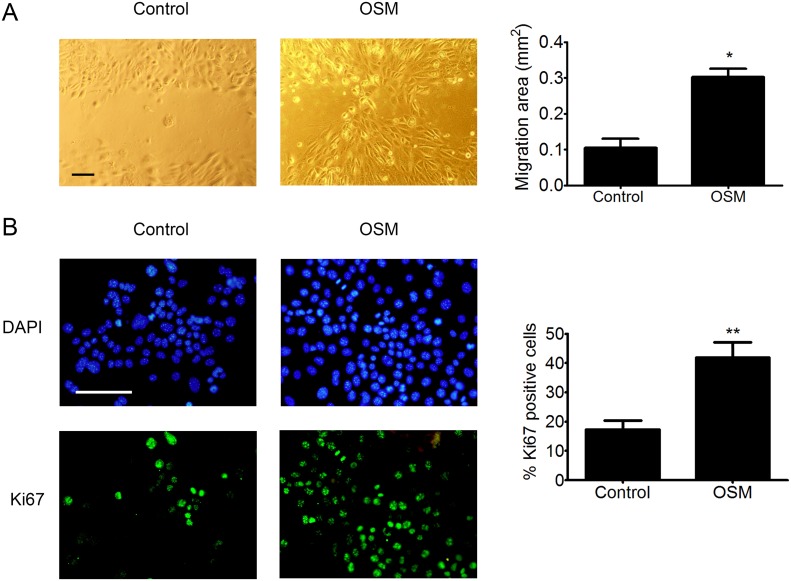
OSM increases the migration and proliferation o PDVC57 cells **(A)** *In vitro* wounds were made in cultures of mitomycin-treated PDVC57 cells and the cells cultured for a further 48 h, with or without OSM, (10 ng/mL). Cell migration into the wounded area was assessed as described in Materials & Methods. Scale bar: 100 μm. Data are presented as the mean ± SEM of the migration area (mm^2^) from three independent experiments. Mann-Whitney test, ^*^p < 0.05. **(B)** PDVC57 cells were cultured, with or without OSM (10 ng/mL), for 48,h. Cells were fixed and stained with an anti-Ki67 antibody (green) and the cell nuclei stained with DAPI (blue). Scale bar: 100 μm. Data are represented as the mean ± SEM of the percentage of Ki67^+^ cells from three independent experiments. Mann-Whitney test, ^*^p < 0.05, ^**^p < 0.01.

Based on the transcriptomic modifications induced by OSM, we then studied the effect of OSM on PDVC57 cell migration and proliferation. We found that OSM increased the migratory capacity of PDVC57 cells after 48 hours of treatment relative to non-stimulated cells in an *in vitro* wound-healing assay. Quantification of the cells in the wounded area showed a three-fold increase in the migration of OSM-stimulated PDVC57 cells (Figure 4A). The percentage of Ki67^+^ cells also significantly increased after 48 hours of OSM treatment, reflecting PDVC57 cell proliferation (Figure 4B).

### SCC development in mice is associated with neutrophil-mediated OSM production

We studied the role of OSM on cSCC *in vivo* by subcutaneously injecting PDVC57 cells into the backs of WT mice. cSCC developed rapidly in 80% of the mice and reached a 2-cm^3^ volume five weeks after engraftment, necessitating euthanasia of the mice, according to the regulations concerning the ethical treatment of animals.

**Figure 5:**
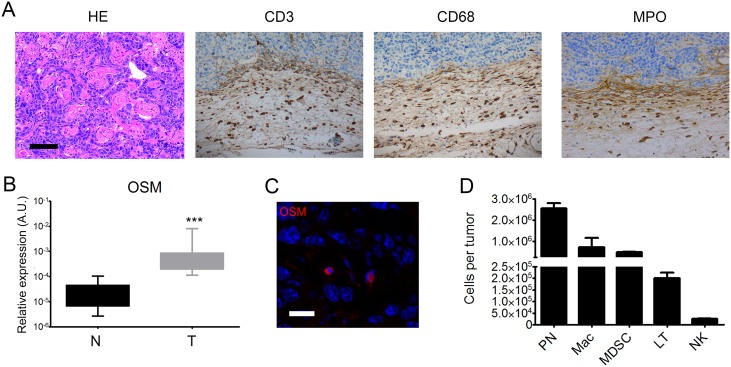
Mouse tumors are moderately differentiated cSCC associated with OSM overexpression by infiltrating neutrophils **(A)** Mouse cSCC sections were fixed, embedded in paraffin, and stained with H&E or an anti-CD3, anti-CD68, or anti-MPO antibody. Scale bar: 100 μm. **(B)** Relative OSM mRNA expression was measured by real-time qPCR in tumors (T) (n = 25) and normal skin (N) (n =2 5). Data are presents as min to max box plots of relative mRNA expression (A.U.). Mann-Whitney test, ^*^p < 0.05, ^**^p < 0.01, ^***^p < 0.001. **(C)** 8-μm frozen sections of tumors from WT mice were fixed in 4% paraformaldehyde and stained with an anti-OSM antibody (R&D system, MAB4951) at 1:50 followed by staining with a goat anti-rat-IgG-AF555 secondary antibody (Thermofisher, A-21434) 1:100. Cell nuclei were stained with TOPRO (Invitrogen). Image acquisition was performed with an Olympus FV1000 confocal microscope using FlowView software (ImageUP platform, University of Poitiers). Scale bar: 20 μm. **(D)** Tumor cells and infiltrating tumor cells were isolated from WT mice. Cells were stained with anti-CD45-V500, anti-CD3-BV421, anti-Ly6G/C-FITC, anti-F4/80-PE, anti-CD68-PerCP-Cy5.5, or antiNK1.1-PE-Cy7. Data are presented as the mean ± SEM of total counts of PN: polynuclear neutrophils, MDSC: myeloid-derived suppressor cells, macrophages, T cells, and NK cells per tumor from three independent experiments.

Histological examination confirmed the development of cSCC, which were moderately differentiated. All tumors destroyed the *panniculus carnosus* and reached the fat tissue underneath. Keratinization was present in 47% of cases. Two tumors displayed tumoral embolus and one, perineural invasion. Inflammatory infiltrates were predominantly present in the invading edge of the tumors and contained lymphocytes, macrophages, and polymorphonuclear cells (Figure 5A). Polymorphonuclear cells were also present in necrotic areas within the tumor. These characteristics were consistent with those observed in human epidermoid carcinomas.

RT-qPCR analysis showed higher OSM expression in SCC than normal skin of WT mice (Figure 5B), as we previously reported for human tumors. We analyzed the cSCC by immunofluorescence to identify the OSM-producing cells. Fluorescent cells with segmented nuclei were present in the tumor stroma, suggesting OSM production by neutrophils (Figure 5C). Flow cytometry analysis after tumor dissociation showed that murine cSCC were mainly infiltrated by neutrophils, macrophages, and myeloid-derived suppressor cells (MDSC), as well as T cells and NK cells (Figure 5D). We never detected OSM in keratinocyte tumor cells.

### cSCC development is reduced in OSM-deficient mice

**Figure 6:**
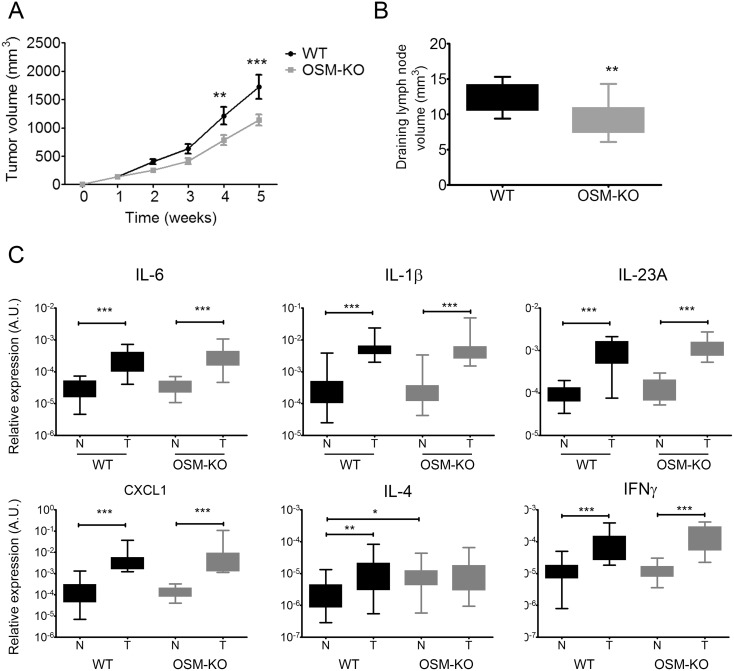
cSCC development is reduced in OSM-deficient mice **(A)** OSM-KO mice and their WT littermates were subcutaneously injected with 1.10^6^ PDVC57 cells and tumor volume was measured each week using a digital caliper. Data are presented as the mean ± SEM tumor volume (mm^3^) from five independent experiments, Two-way ANOVA test with Bonferroni post-tests, ^**^p < 0.01, ^***^p < 0.001. **(B)** The size of draining lymph nodes were measured under the microscope using a ruler and the volume calculated according to the formula: length x width x thickness. **(C)** Relative mRNA expression was measured by real-time qPCR in tumors (T) (n = 25) and normal skin (N) (n = 25) of OSM-KO mice and WT littermates. Data are presented as min to max box plots of relative mRNA expression (A.U.). Kruskall-Wallis test with Dunn's Multiple Comparison Test, ^*^p < 0.05, ^**^p < 0.01, ^***^p < 0.001.

We investigated the involvement of OSM in tumor development by comparing the size of cSCC tumors between OSM-KO mice and their WT littermates. The tumors of OSM-KO mice were smaller than those of WT mice from week 2 and the difference was significant by weeks 4 and 5, approximately 30% (Figure 6A). Moreover, we collected and measured the draining lymph nodes and observed a smaller volume for those from OSM-KO mice than those from their WT littermates (Figure 6B). Histological parameters were similar in terms of differentiation, keratinization, and inflammatory infiltrates. There was no perineural infiltration and only one tumoral embolus was observed. However, necrotic areas (6.8% *vs* 9.3 % of all tumoral areas) and mitotic counts (43.2 *vs* 50.6 mitoses per 10 high power fields) of OSM-KO mice were slightly less than those of WT mice.

Flow cytometry analyses showed neither qualitative nor quantitative differences between the immune infiltrates of WT and OSM-KO mice (data not shown). We further analyzed cytokine expression by RT-qPCR of tumors and normal-associated skin of WT and OSM-KO mice. Aside from greater expression of OSM transcripts in tumors than the normal skin of WT mice (Figure 5B), mRNA expression of the pro-inflammatory cytokines IL-6, IL-1β, and IL-23A was also up-regulated (Figure 6C), whereas IL-17A and IL-22 transcripts were not detected. TNFα mRNA expression was not modulated (data not shown) and CXCL1 was more highly expressed in tumors than normal skin. Both IFNγ and IL-4 mRNA were more highly expressed in tumors than normal skin (Figure 6C), as was that of the immunosuppressive cytokine transcripts IL-10 and TGFβ (data not shown). We did not detect any OSM transcripts in the tumors of OSM-KO mice, demonstrating that the injected PDVC57 cells did not produce OSM (data not shown). Moreover, the cytokine signature was the same between normal skin and cSCC of OSM-KO mice and their WT littermates, except for IL-4 mRNA levels, which were higher in the normal skin of the OSM-KO mice than that of their WT littermates (Figure 6C).

### OSM is involved in M2 polarization

**Figure 7:**
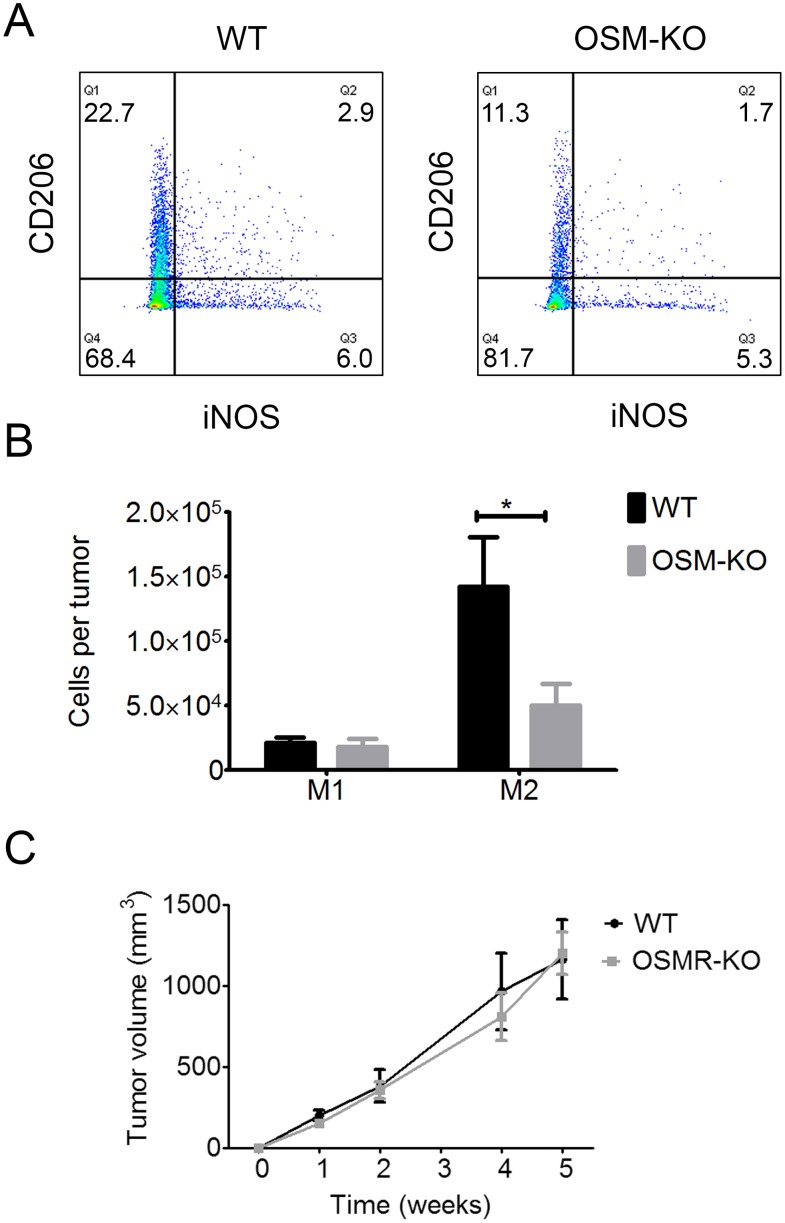
M2 macrophage polarization is reduced in SCC from OSM-KO mice and cSCC development is similar in WT and OSMR-KO mice **(A)** Tumor cells and infiltrating cells were isolated from OSM-KO mice and their WT littermates. Cells were stained with anti-CD45 V500, anti-CD11b AF488, anti-CD68 PerCPCy5.5, anti-CD206-BV421 or anti-iNOS-APC antibody and analyzed by flow cytometry. **(B)** Data are presented as the mean ± SEM of the percentage of M1 (iNOS^+^/CD206^-^) and M2 (iNOS^-^/CD206^+^) macrophages among CD45^+^ cells per tumor from four independent experiments, Two-way ANOVA test with Bonferroni post-tests, ^*^p < 0.05. **(C)** OSMR-KO mice and their WT littermates were subcutaneously injected with 1.10^6^ PDVC57 cells and tumor volume measured each week using a digital caliper. Data are presented as the mean ± SEM tumor volume (mm^3^) from two independent experiments, Two-way ANOVA test with Bonferroni post-tests.

The overall transcriptomic cytokine signature of the microenvironment was not modified by the absence of OSM. However, recent studies demonstrated OSM involvement in M2 macrophage polarization (36, 37). Thus, we assessed M2 polarization in cSCC by flow cytometry. The percentage of CD206^+^iNOS^-^ M2 macrophages was lower in the cSCC of OSM-KO mice than those of WT mice (Figure 7A), as well as the total number of M2 macrophages in the tumor, which was also significantly lower (65%) for OSM-KO than WT mice (Figure 7B), whereas there was no difference in the M1 macrophage count.

### OSM exerts most of its pro-tumoral activity directly on tumor cells

We examined whether OSM exerts a direct pro-tumoral activity on cancer cells or has an indirect effect through the activation of cells present in the peritumoral environment by assessing tumor growth in mice deficient for the OSMRβ subunit (OSMR-KO) and their WT littermates. After five weeks of development, the tumor volume was the same for both OSMR-KO mice and their WT littermates, suggesting a direct role of OSM on OSMR-expressing cancer cells to promote tumor development (Figure 7C).

## DISCUSSION

A decade ago, OSM was reported to directly target keratinocytes *in vitro* by inducing epidermal hyperplasia of reconstructed human epidermis [27] and to modulate the expression of genes involved in differentiation, proliferation, innate immunity, angiogenesis, motility, and tissue remodeling *in vivo* [42]. The properties and overexpression of OSM in human cSCC tumors prompted us to investigate its involvement in cSCC development. Indeed, recent studies have suggested a contribution of OSM in cancer development. This cytokine was first called ″oncostatin″, due to its protective effects in melanoma; however, direct deleterious or indirect effects on cancer development have been further reported. Indeed, OSM increases cell motility and the invasiveness of cervical squamous carcinoma cells [43] and induces the proliferation of prostate cancer 22Rv1 cells [36]. It activates migration and invasion of endometrial cancer cells through the STAT3 signaling pathway. OSM can also act on the tumor microenvironment and promotes cancer development through the induction of M2 macrophage polarization in breast and lung cancer [38, 39].

In addition, the expression of other pro-inflammatory cytokines, such as IL-1, IL-6, or IL-22, produced during the tumor-induced inflammatory response, are also likely to be integrated in this network [6]. Indeed, we previously demonstrated synergism between OSM, IL-17A, IL-1, TNFα, and IL-22 to induce skin inflammation both *in vitro* and *in vivo* [28, 29, 31]. These pro-inflammatory cytokines were all found to be upregulated in human and mouse cSCC, except IL-22, although Nardinocchi *et al.,* reported IL-22 expression by T cells associated with pro-tumoral effects in cSCC [23]. The overall cytokine expression in our cohort favors a type 1 profile, with a strong correlation between OSM, IL-1β, and IL-8 expression, suggesting a coordinated cytokine network. Indeed, we previously showed a link between OSM and IL-1β activity in inflammatory skin. These cytokines induced an inflammation state related to the pathogenesis of patients with hypertensive leg ulcer. OSM and IL-1β also synergistically promoted acanthosis, a diffuse epidermal hyperplasia, on reconstructed epidermis *in vitro* and *in vivo* [31]. A correlation between the expression of OSM and the neutrophil chemotactic factor IL-8 suggests that OSM is associated with neutrophils. In 1999, Grenier *et al*. showed that a combination of LPS and CSF2 induced neutrophil-derived OSM production *in vitro* and proposed that OSM production is involved in the modulation of local inflammation [44].

However, Pettersen *et al.* showed overexpression of IFNγ and IL-4 in human cSCC, suggesting the presence of both type 1 and type 2 immune responses [17]. However, this study focused on the cytokine expression of T cells and macrophages isolated from tumors rather than that of the global tumor microenvironment, which may explain the observed discordance.

We further explored the possibility of a direct action of OSM on malignant keratinocytes. We used the transformed-keratinocyte murine cell line, PDVC57, to show that OSM activates the MAPK and JAK/STAT3 pathways, as previously described in humans for normal keratinocytes [27, 33] and other OSM-stimulated cancer cells [36, 45]. OSM modulates the expression of genes involved in the immune response, such as BCL3, IL13RA1, and LCN2. Some of these genes could be involved in tumor development such as LCN2. This gene induces migration and invasion of esophageal squamous cell carcinoma cells [46]. Recently, Jung et al., found that LCN2, an iron transporter enhances tumor development by making the microenvironment more permissive through macrophages and iron trafficking [47]. This protein is also a marker for dysregulated keratinocyte differentiation in human skin [48]. The expression of these genes is also regulated by OSM in normal murine epidermal keratinocytes (unpublished data). In addition, OSM-enhanced migration and proliferation of PDVC57 cells is in accordance with a previous *in vitro* study on the migration of normal keratinocytes [27] and that of different types of cancer cells, such as prostate cancer 22Rv1, cervical squamous cell carcinoma, and myeloma [36, 43, 49]. Overall, these results suggest the direct involvement of OSM in cSCC tumoral processes.

Nonetheless, the question of the effect of OSM on tumor development *in vivo* is still unresolved. We detected high OSM mRNA expression by cSCC in both humans and mice. If in mouse tumors, OSM is produced mainly by tumor-infiltrating neutrophils, we lack validated tools to detect OSM production in human tumors. However, OSM is known to be produced *in vitro* by neutrophils from healthy donors [49] and those isolated from the respiratory epithelium of patients with chronic rhinitis [50]. In the context of cancer development, breast cancer cells have been shown to stimulate OSM production by neutrophils, with potential pro-tumoral activity [51]. This finding is consistent with those of other studies reporting type 1 and type 2 polarized neutrophil subsets [52, 53], which are involved in tumor regression or tumor development, respectively, similarly to type 1 and type 2 T cells or macrophages. However, compared to human tumors, neutrophils are more present than T cell and macrophages in our mouse cSCC model. This could be explained by the rapid tumor development, which induces an important tumor necrosis ensuing neutrophil infiltration. If neutrophils are the main OSM-producer in our mouse cSCC model, we did not exclude a potential production by T cell and/or macrophages.

We analyzed and compared cSCC development of OSM-KO mice to that of WT littermates. The reduced size of tumors in OSM-KO mice demonstrates its involvement in tumor development. The expression of other STAT3-signalling cytokine, such as IL-6, could explain the absence of total tumor regression and the similar cytokine expression and immune infiltration profiles between OSM-KO mice and their WT littermates. Moreover, we analyzed mRNA relative expression of *in vitro*-OSM-regulated genes and we found no significant differences between tumors developed on OSM-KO mice and their WT littermates. Interestingly, there were a number of similarities between human and murine cSCC expression profiles. We analyzed macrophage polarization because of the major role of macrophages in tumor development [10] and OSM involvement in macrophage polarization in other cancers. OSM promoted M2 polarization in our cSCC mouse model, as observed by Tripathi *et al.* in breast cancer [38]. However, there was no difference in tumor growth between OSMR-KO mice and their WT littermates, suggesting that the main pro-tumoral effect of OSM in this model is exerted directly on tumor cells.

In summary, we demonstrated that neutrophil-derived OSM production may directly induce cSCC proliferation and modify the tumor microenvironment toward M2 polarization, promoting tumor progression. In addition, our results show that OSM overexpression in our cSCC mouse model has a direct pro-tumoral effect, thus opening new therapeutic approaches that target this cytokine [54]. It would be informative to test the therapeutic potential of anti-OSM neutralizing antibodies in the development of cSCC, as antibody-based therapeutics against cancer are highly successful in the clinic.

## MATERIALS AND METHODS

### Patients

Twenty-two adults with a clinical diagnosis of cSCC were included in this study. All diagnoses were confirmed by histological analysis of the excised tumors. After surgery, biopsies of the tumoral lesion (SCC) and a macroscopically healthy area at the tumor margin (peritumoral SCC or P SCC) were obtained. The remaining material was fixed in formalin and subjected to the normal procedures of surgical pathology. Normal skin samples were obtained from surgical samples of abdominoplasty or breast reduction surgery and were used as controls. Samples were immediately frozen in liquid nitrogen for RNA isolation. Samples were kept frozen at -80°C in the Poitiers Biological Resources Center (BRC BB0033-00068). Clinical information, such as age, gender, medical history of organ transplantation, tumor location, tumor size, recurrence, metastasis, and medical follow-up were recorded.

The Poitiers University Hospital Ethics Committee approved the study design (CPP Ouest III - DC-2008-565) and the study followed the Declaration of Helsinki Protocols. All participants in the study gave their informed written consent.

### Histological analysis

All tumors were fixed in formalin and sectioned by a trained dermatopathologist, according to standard procedures for skin tumors. All tissue sections were embedded in paraffin and sequential 3-μm sections obtained and colored by hematoxylin, eosin, and safran (HES). Several standard histological criteria were recorded, such as tumor differentiation state, keratinization, perineural invasion, embolus, Clark level, epidermal or tumoral ulceration, and safety margin size. Infiltration depth was measured using an oculometer, according to the Breslow recommendations for melanoma.

### Cell culture

Normal murine epidermal keratinocytes (NMEK) were isolated and cultured as previously described [30]. PDVC57 cells were obtained from Dr Quintanilla (Madrid, Spain). They were initially isolated from a tumor induced in C57Bl/6 mice by subcutaneous injection of newborn mouse keratinocytes that were chemically transformed with DMBA and called PDV cells [41]. PDVC57 cells were cultured in DMEM/F-12 medium supplemented with GlutaMAX™ (Invitrogen), 10% Fetal Bovine Serum (FBS) (Invitrogen), and 1% Penicillin/Streptomycin (P/S) (Invitrogen), at 37°C and 5% CO_2_. At 80% confluency, cells were starved for 24 h in DMEM/F-12 medium with 0.1% FBS and 1% P/S. The cells were then stimulated, or not, with recombinant murine OSM (R&D Systems) at a final concentration of 10 ng/mL.

### Animals and *in vivo* murine SCC development

Mice deficient for OSM (OSM-KO) and OSMRβ (OSMR-KO) were obtained from Pr. A. Miyajima (Tokyo, Japan) [55, 56]. KO mice and their wild-type (WT) littermates were housed in the same facility, under pathogen-free conditions, and maintained on a 12-h light/dark cycle, with food and water *ad libitum*. All protocols were approved by the regional ethical committee (C2EA-84) under agreement number 2015120717501373.

For SCC development, PDVC57 cells were cultured to 80% confluency and dissociated by trypsin-EDTA digestion (Invitrogen) for 10 min at 37°C. The cells were washed three times with 1X Phosphate Buffered Saline (PBS) (Invitrogen) and suspended in physiological serum at a cell density of 10×10^6^ cells/mL. Mice were anesthetized by inhalation of 2.5% isoflurane (Forene, Abbott France). PDVC27 cells (1×10^6^/injection) were subcutaneously injected into the shaved back of 6 to 8-week-old OSM-KO and WT males. Mice were monitored and weighed three times a week for clinical signs of health status indicators. Tumor volumes were determined once a week. The length (the longest dimension) and width (the distance perpendicular to the length) of each tumor were measured with a digital caliper. The formula used to determine tumor volume was V(volume) = π/6^*^ L(length)^*^W^2^(width) [57].

After five weeks, mice were euthanized by cervical dislocation and the tumor and contralateral healthy skin collected and rapidly frozen in liquid nitrogen for mRNA quantification and immunofluorescence analyses, embedded in paraffin for standard histology and IHC, or conserved in culture media for flow cytometry analyses.

### RNA isolation and real-time quantitative RT-PCR

Tissue dissociation of human samples was performed mechanically in liquid nitrogen that of mouse samples using a gentleMACS dissociator (Miltenyi Biotec). Total RNA was isolated using a NucleoSpin RNA kit (Macherey-Nagel) according to the manufacturer's instructions. RNA was reverse-transcribed with Superscript II reverse transcriptase (Invitrogen) and the transcripts amplified and quantified using the LightCycler-FastStart DNA Master Plus SYBR Green I kit on a LightCycler 480 instrument (Roche Diagnostics).

Sense and antisense oligonucleotides were designed using Primer-Blast (NCBI) and purchased from Eurogentec. The mRNA expression of the human samples was normalized against that of two housekeeping genes, glyceraldehyde-3-phosphate dehydrogenase (GAPDH) and beta-actin (ACTB). The mRNA expression of the mouse samples was also normalized against that of two housekeeping genes, glyceraldehyde-3phosphate dehydrogenase (GAPDH) and beta-2-microglogulin (B2M).

### Western blot analysis

For STAT3 and ERK1/2 phosphorylation studies, PDVC57 cells were stimulated, or not, with 10 ng/mL OSM for various times and lysed in SDS sample buffer containing Tris-HCl 100 mM pH 6.8, 2% SDS, 5% 2-mercaptoethanol, 2.5% glycerol, and 0.1% bromophenol blue. The lysates were separated by SDS-PAGE, transferred onto a polyvinylidene difluoride membrane (Amersham biosciences), and probed with anti-Phospho-STAT3 (Cell Signalling, 9145S, 1:2000), anti-Phospho-ERK1/2 (SantaCruz, sc-16982-R, 1:1000), antiSTAT3 (SantaCruz, sc-482, 1:1000), or anti-ERK1/2 (SantaCruz, sc-93, 1:1000) overnight, revealed with a peroxidase-conjugated goat anti-rabbit IgG (Sigma, A0545, 1:10000), and visualized by chemiluminescence using a LAS-3000 imager (Fujifilm). Signal intensities were measured using ImageJ software (National Institutes of Health).

### Affymetrix assay

Total RNA was isolated from PDVC57 cells cultured with or without rOSM for 6 h or 24 h and used for microarray analysis. Briefly, RNA was extracted using the NucleoSpin RNA kit (Macherey-Nagel), according to the manufacturer's instructions. Probes were prepared and hybridized to the Affymetrix GeneChip™ Mouse Gene 2.1 ST Array Plate (Affymetrix) as recommended by the manufacturer. Data were obtained from measurements of the relative signal strength for probes containing ~30,000 transcripts. Data were submitted to the Gene Expression Omnibus Database (GSE111191) and to the Database for Annotation, Visualization and Integrated Discovery (DAVID) for clustering analysis [58].

### Wound healing assay

PDVC57 cells were cultured until the formation of a monolayer. After 24 h of starvation, cells were treated with 10 μg/mL mitomycin C (Sigma) for 2 h. A scratch was created with a 200-μl tip on the monolayer and cells were incubated with or without OSM (10 ng/mL) for 48 h. The migration capacity of PDVC57 cells to fill the gap created by the scratch was measured at baseline and after 48 h of treatment by capturing five images for each well (CellSense Software, Olympus) and measuring the surface of the scratch using ImageJ software.

### Proliferation assay

PDVC57 cells were seeded on coverslips in a four-well plate. After 24 h of starvation, cells were incubated with or without 10 ng/mL rOSM for 48 h. The cells were then fixed in acetone/methanol (20/80) for 20 min and permeabilized with 1X PBS, 5% bovine serum albumin (BSA) (Sigma) and 0.3% Triton-X100 (Sigma) before incubation with an anti-Ki67 mAb (Dako, M7249, 1:100) and then a FITC-conjugated donkey anti-rat IgG (Jackson & ImmunoResearch, 712-096-153, 1:100). Cell nuclei were detected with DAPI (Sigma, 1:1000) and the number of Ki67^+^ cells counted in three non-overlapping fields by fluorescence microscopy (Olympus).

### Immunohistochemistry and immunofluorescence

For IHC assessment, a tissue micro-array was constructed using cores of 0.6 mm in diameter. Three zones were used for each tumor: intratumoral, invasion front, and peritumoral. Three cores were used for each zone. Peritumoral zones were not obtained for two tumors. Sequential 3-μm sections were cut and colored by HES or used for immunohistochemistry.

IHC was also performed on tissue sections from formalin-fixed paraffin-embedded tissue blocks from WT and OSM-KO mice. Serial sections of 3 μm were cut. Immunohistochemistry was performed using a BenchMark automated staining system (Ventana Medical System, Tucson, AZ) for CD3 (Dako, A0452, 1:200), CD68 (Ventana, 790-2931, ready to use), and MPO (Dako, IS511, ready to use). Images were taken using a camera fixed to the microscope (Leica Application suite, version 4.4). Quantification of inflammatory cells was performed using assisted counting software (Visilog Noesis).

For immunofluorescence studies, tumor sections were fixed in acetone/methanol (20/80) and stained with anti-OSM (R&D system, MAB4951, 1:50), followed by staining with a goat anti-rat-IgGAF555 secondary antibody (Thermofisher, A-21434, 1:100). Cell nuclei were stained with TOPRO (Invitrogen). Image acquisition was performed with an Olympus FV1000 confocal microscope using FlowView software (ImageUP platform, University of Poitiers).

### Cell preparation and flow cytometry

Tumor cell suspensions were obtained after mechanical and enzymatic disruption and filtration using a mouse tumor dissociation kit (Milteniy Biotec), according to the manufacturer's instructions. To study cytokine production, cells were stimulated with a cell stimulation cocktail (ebiosciences) and Golgi Plug (BD Biosciences) for 5 h. Then, the cells were incubated with Fc Block (BD Biosciences, 553142) for 15 min at 4°C and then 30 min at RT with Zombie NIR (BioLegend, 423105). Cells were stained with various combinations of the following antibodies: V500-conjugated anti-CD45 (BD Horizon, 561487), AF488-conjugated anti CD11b (BioLegend, 101217), BV421-conjugated anti-CD206 (BioLegend, 141717), BV421-conjugated anti-CD3 (BD Horizon, 564008), PE-conjugated anti-F4/80 (BioLegend, 123110), FITC-conjugated anti-Ly6G/C (BD Pharmingen, 553127).

For intracellular staining, cells were permeabilized with Cytofix/Cytoperm and labelled with APC-conjugated anti-iNOS (ebiosciences, 17-5920) and PerCP/Cy5.5-conjugated anti-CD68 (BioLegend, 137010). Data were collected with a FACSVerse instrument and analyzed using FlowJo software version 10 (FlowJo, LLC).

### Statistics

Statistical analyses were conducted using the Kruskall-Wallis test with Dunn's multiple comparison test, the Mann-Whitney test, or two-way ANOVA with Bonferroni post-tests (GraphPad Prism software, version 5.0). Results are presented as min to max box plots, mean ± SEM, or scatter-plot graphs. *P* values < 0.05 were considered to be significant.
